# Costing Framework for International Health Regulations (2005)

**DOI:** 10.3201/eid1807.120191

**Published:** 2012-07

**Authors:** Rebecca Katz, Vibhuti Haté, Sarah Kornblet, Julie E. Fischer

**Affiliations:** George Washington University, Washington, DC, USA (R. Katz);; and Stimson Center, Washington (V. Haté, S. Kornblet, J.E. Fischer)

**Keywords:** International Health Regulations, disease surveillance, capacity building, laboratory capacity, response, preparedness, costing

## Abstract

The revised International Health Regulations (IHR [2005]) conferred new responsibilities on member states of the World Health Organization, requiring them to develop core capacities to detect, assess, report, and respond to public health emergencies. Many countries have not yet developed these capacities, and poor understanding of the associated costs have created a barrier to effectively marshaling assistance. To help national and international decision makers understand the inputs and associated costs of implementing the IHR (2005), we developed an IHR implementation strategy to serve as a framework for making preliminary estimates of fixed and operating costs associated with developing and sustaining IHR core capacities across an entire public health system. This tool lays the groundwork for modeling the costs of strengthening public health systems from the central to the peripheral level of an integrated health system, a key step in helping national health authorities define necessary actions and investments required for IHR compliance.

## Abstract 2

Costs can be estimated by identifying functional pathways toward achieving all 8 core capacities and global indicators.

In 2005, the member states of the World Health Organization (WHO) recognized the need to overhaul international public health cooperation, and they revised the International Health Regulations (IHR). The IHR (2005) focus on strengthening capabilities for confronting all potential public health emergencies of international concern when and where they occur. The 194 states parties made a commitment to develop core capacities to detect, assess, report, and respond to any public health event that might have international effects, regardless of type or origin of the event. The IHR (2005) also conferred new responsibilities on WHO and the global health community to share resources, information, and expertise to help nations prepare for and respond to public health events (*1*).

The WHO checklist and indicators for monitoring progress in the development of IHR core capacities by states parties, also known as the IHR Monitoring Framework, details 8 core capacities plus activities at points of entry that must be developed to fully implement the IHR (Table 1) (*2*). The IHR Monitoring Framework, first published in 2010, also defines country-level indicators within each core capacity. The regulations and the framework describe the core capacities needed for functional implementation of the IHR (2005) but leave flexibility for nations to determine how best to structure and develop these capacities (*3*).

**Table 1:** Summary of 2010 World Health Organization IHR Monitoring Framework*

Core capacity	Component	Country-level indicator
National legislation, policy, and financing	National legislation and policy	Laws, regulations, administrative requirements, policies, or other government instruments in place are sufficient for implementation of obligations under IHR.
	Financing	Funding is available and accessible for implementing IHR (including developing core capacities).
Coordination and NFP communications	IHR coordination, communication, and advocacy	A mechanism is established for the coordination of relevant sectors in the implementation of IHR.
		IHR National Focal Point functions and operations are in place as defined by the IHR (2005).
Surveillance	Indicator-based, or routine, surveillance (also referred to as structured surveillance, routine surveillance, and surveillance for defined conditions)	Indicator-based, routine, surveillance includes the early warning function for the early detection of public health events.
	Event based surveillance established	Event-based surveillance is established.
	Surveillance overview of information on IHR-related hazards (situation awareness)	A coordinated mechanism is in place for collecting and integrating information from sectors relevant to IHR
Response	Rapid response capacity	Public health emergency response mechanisms are established.
	Case management	Case management procedures are established for IHR-relevant hazards.
	Infection control	Infection prevention and control is established at national and hospital levels.
	Disinfection, decontamination, and vector control	A program for disinfection, decontamination, and vector control is established.
Preparedness	Public health emergency preparedness and response	Multihazard national public health emergency preparedness and response plan is developed.
	Risk and resource management for IHR preparedness	Public health risks and resources are mapped.
Risk Communication	Policy and procedures for public communications	Mechanisms for effective risk communication during a public health emergency are established.
Human Resources	Human resource capacity	Human resources are available to implement IHR core capacity requirements.
Laboratories	Laboratory diagnostic and confirmation capacity	Laboratory services are available and accessible to test for priority health threats.
		Influenza surveillance is established.
	Specimen collection and transport	System for collection, packaging, and transport of clinical specimens is established.
	Laboratory biosafety and biosecurity	Laboratory biosafety/biosecurity practices are in place.
	Laboratory-based surveillance	Laboratory data management and reporting is established.
Points of Entry	Surveillance at points of entry	Effective surveillance is established at points of entry.
	Response at points of entry	Effective response at points of entry established.

*IHR, International Health Regulations; NFP, National Focal Point. Data from (*2*).

The IHR also direct countries to strengthen and integrate existing systems for public health surveillance and response, rather than to create new, vertical programs. Various national approaches to IHR implementation have emerged, depending on factors such as the sophistication of preexisting systems and infrastructure, past and present objectives of health ministries and their external partners, availability of resources, architecture of health systems, and strength of regional commitments to health cooperation and coordination. Examples of the latter are the Integrated Disease Surveillance and Response strategy previously adopted by the WHO Regional Committee for Africa and shared standards developed through a Latin American subregional trade alliance (*4**–**7*). Two WHO regional offices, Southeast Asia Regional Office (SEARO) and Western Pacific Regional Office (WPRO), collaboratively developed the Asia Pacific Strategy for Emerging Diseases, providing a framework for coordinated approaches to rapid disease detection and public health emergency responses across sectors, countries, and regions (*8*).

Even with regional support, achieving the IHR core competencies is challenging for many nations at high risk for epidemic-prone or emerging infectious disease outbreaks and other public health crises. Member states initially agreed to implement IHR (2005) by June 2012, but a substantial proportion will clearly need at least one 2-year extension. Under Article 44 of the IHR, nations agreed to collaborate on developing and maintaining the public health capacities for IHR implementation by providing technical, logistical, and financial assistance to developing nations. The flexibility of the IHR framework, which enables national leaders to interpret the IHR requirements through mechanisms that are sensitive to local and regional contexts, makes it challenging to marshal such assistance effectively. The decision to measure IHR core capacity development in terms of functional outcomes rather than specific activities means that there could be 194 distinct but equally valid national approaches to fulfilling IHR (2005) obligations. Consequently, many nations that could use help with IHR implementation are still in the process of identifying opportunities for cooperative capacity building with external partners, often without information on how much it will cost to implement their national IHR action plans.

We describe steps for estimating the costs of achieving IHR (2005) implementation in countries with different economic climates by first identifying essential inputs. We identified functional pathways for implementing the 8 core capacities and actions at points of entry identified in the WHO 2010 IHR Monitoring Framework, on the basis of current and planned actions in 6 Southeast Asian case-study countries at different levels of economic and health systems development. We used this to develop a representative IHR implementation strategy to serve as a framework for a preliminary estimate of fixed and operating costs associated with developing and sustaining IHR core capacities across an entire public health system.

## Methods

### Case-Study Countries

To develop an initial costing framework for IHR implementation, we sought case-study countries that could provide examples of field-tested strategies and practices in the 8 IHR core capacities and at points of entry, along with associated costs. On the basis of geographic proximity, recent responses to emerging infectious diseases of public health significance, economic development levels, and accessibility of financial and policy information, we identified 6 case-study countries in Southeast Asia: 1 low-income (Cambodia), 3 lower-middle income (Lao People’s Democratic Republic, Vietnam, and Timor-Leste), and 2 upper-middle income (Malaysia and Thailand) (*9*). These 6 countries fall into the SEARO and WPRO areas, which share the Asia Pacific Strategy for Emerging Diseases capacity-building strategy (*8*).

### Core Capacities Matrix

The 2010 WHO IHR Monitoring Framework identified 20 country-level indicators that states parties could use to assess IHR core capacity development (Table 1). The framework described levels of capability that could be used to evaluate progress toward each indicator, categorizing capabilities as prerequisites/foundational (level <1), inputs and processes (level 1), outputs and outcomes (level 2), and additional (level 3). Framework guidance specified that for all indicators to meet IHR requirements, countries must successfully demonstrate the attributes at levels 1 and 2.

For each country-level indicator, we identified specific activities and resources that could operationally achieve levels 1 and 2 attributes. Identification involved a 2-step process: 1) determining whether a technical standard exists for achieving each country-level indicator and 2) mapping activities and strategies among the case-study countries to the IHR Monitoring Framework.

To identify standards for building and sustaining the 8 core capacities, we reviewed guidance published by WHO and its regional offices, accrediting and professional organizations, and the US Centers for Disease Control and Prevention; consensus recommendations developed by expert working groups; and peer-reviewed publications, supplemented by additional input from subject matter experts in relevant disciplines.

To describe capabilities, activities, tools, and processes identified by decision makers in each case-study country as relevant to IHR core capacities, we reviewed published and unpublished government documents (e.g., legislation; regulations; national strategies; operational and programmatic guidance; training materials; self-assessments; proposed and enacted budgets; and plans for developing, strengthening, or maintaining IHR core capacities, pandemic preparedness, public health or emergency medical preparedness, indicator- and event-based surveillance, and laboratory systems) and materials prepared with or for development partners, technical partners, and nongovernmental stakeholders in each country. We supplemented the literature review through interviews with governmental and nongovernmental stakeholders in case-study countries. To determine requirements for diagnostic testing capabilities, we derived a priority disease list comprised of the endemic, epidemic-prone, and emerging infectious diseases specifically cited as always notifiable by the IHR (2005) Annex 2 reporting algorithm plus those appearing on >3 case-study country priority disease lists.

We mapped the activities and strategies identified through the reviews of technical guidance and case-study country activities to specific country-level indicators in the IHR Monitoring Framework, creating an operating core capacities matrix. To identify the practices and attributes common to some or all case-study countries for each core capacity, we compared these activities and strategies, distilling the strategies and practices into a representative Southeast Asian country, hereafter referred to as Country X, that has achieved levels 1 and 2 under each country-level indicator. Where no clear consensus emerged on strategies or practices, we selected the national approach that most closely resembled international or regional technical standards.

### Costing Framework

For each activity or capability mapped to a specific country-level indicator in the Country X core capacities matrix, we extrapolated requirements for physical infrastructure (facilities, equipment, utilities), human capabilities (workforce, training, skills, and knowledge), and tools and processes (e.g., diagnostic platforms, materials, reagents, quality control and assurance, reporting systems), building on the foundations of existing public health surveillance costing platforms, such as the Integrated Disease Surveillance and Response SurvCost tool (*10*). Because of the integrated nature of the IHR core capacities, some physical infrastructure, human resources, and tools and processes might contribute to multiple core capacities. We sought to prevent overlap by including such elements only 1 time, under the most immediately relevant indicator.

To develop a preliminary cost estimate for developing and sustaining such infrastructure, human capabilities, and tools and process, we used the following: 1) costs calculated by case-study country government actors for procurement or national budgets; 2) estimates derived with or for international partners; 3) the WHO CHOICE (*CHO*osing *I*nterventions that are *C*ost *E*ffective) database as a source of average salaries, per diem and travel compensation, physical infrastructure, and tradables specific to the subregions of SEARO B and WPRO B, into which the case-study countries fall; and 4) commercial price lists and supply schedules. Because the WHO CHOICE dataset expresses average costs in 2005 international dollars (defined as equivalent to $US in 2005 purchasing power parity), we likewise included or adjusted all costs in 2005 $US. We did not attempt to distinguish between the contributions of public, private, or international actors in mapping the surveillance, response, and laboratory systems for Country X. We thus assumed that the total costs of developing and sustaining each activity or capacity would be the same regardless of the payer. We did not attempt to calculate tariffs or other additional fees specific to each country.

### Assumptions and Limitations

**Figure 1:**
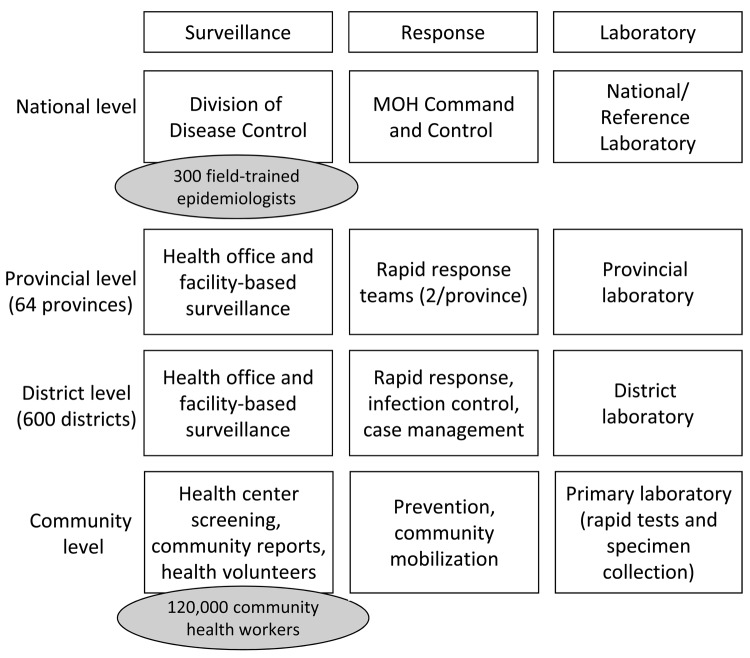
Overview of national public health system for model Southeast Asian country with a population of 60 million. MOH, Ministry of Health.

The Country X template represents a composite of demographic, political, and geographic attributes of 6 low- to middle-income case-study countries in 2 WHO sub-regions (SEARO B and WPRO B). All estimates for Country X assume a population of 60 million persons; 64 provinces with 600 functional districts; and 6 designated points of entry with a Ministry of Health responsible for public health surveillance, response, and laboratory capabilities at the national, provincial, district, and community levels (Figure 1).

Among the case-study countries, national strategies for public health surveillance depend heavily on facility-based surveillance. The reliability and timeliness of facilities-based reporting depend on population access to basic health services with trained health workers at peripheral, intermediate, and central levels. Such services are an absolute prerequisite to IHR implementation but are not explicitly included in the IHR (2005) or associated guidance. Any estimates for costs of public health surveillance and response developed through the framework described here should therefore be considered additional to the costs of developing and sustaining adequate essential health services.

## Results

When we identified practices and strategies in 6 case-study Southeast Asian countries, referenced against regional/global technical standards, that could achieve the functional outcomes specified by each country-level indicator in the IHR Monitoring Framework, the resulting core capacities matrix created a detailed template for fully implementing IHR (2005) in a model Southeast Asian country. We present this matrix as a framework for determining the inputs—physical infrastructure, human capabilities, and tools and processes—required to achieve each core capacity at the peripheral, intermediate, and central levels of the Country X template and for estimating the costs associated with these inputs (Table 2).

**Table 2:** Summary of costs for all 8 International Health Regulations core capacities and ports of entry in Country X

Core capacity	Fixed costs, $US	Operating costs, $US
National legislation, policy, and financing	75,000	0
Coordination and National Focal Point communications	823,102	347,959–88,868
Surveillance	5,261,764	26,238,293–69,606,113
Response	20,480,332	3,981,294–5,215,857
Preparedness	2,889,166	103,726,507–103,786,408
Risk communications	4,389	1,868,869–2,141,939
Human resources	4,389	620,649–653,009
Laboratories	49,619,443	13,742,692–20,057,218
Points of entry	153,062	838,851–1,435,767
Total	79,310,647	151,365,114–203,485,179
Total cost, fixed + operating	Not applicable	230,675,761–282,795,826

### Core Capacity 1: National Legislation, Policy, and Financing

Country-level indicators focus on adoption of budgetary and regulatory frameworks to support IHR implementation. We identified only 1 input with cost implications—support for legal expertise (domestic or external consultants) to review and, as needed, revise national public health laws, estimated at $75,000 (in 2005 $US)—which was based on past consulting costs for revising national regulations with regard to avian and human influenza.

### Core Capacity 2: Coordination and National Focal Point Communications

IHR coordination, communications, and advocacy require designation of an IHR National Focal Point and mechanisms to identify, convene, and coordinate stakeholders in public health surveillance and response across sectors. Inputs include information and communications technologies equipment and services as well as office infrastructure, transportation, and salary support for the individual or office serving as National Focal Points (Technical Appendix Table 1).

### Core Capacity 3: Surveillance

**Figure 2:**
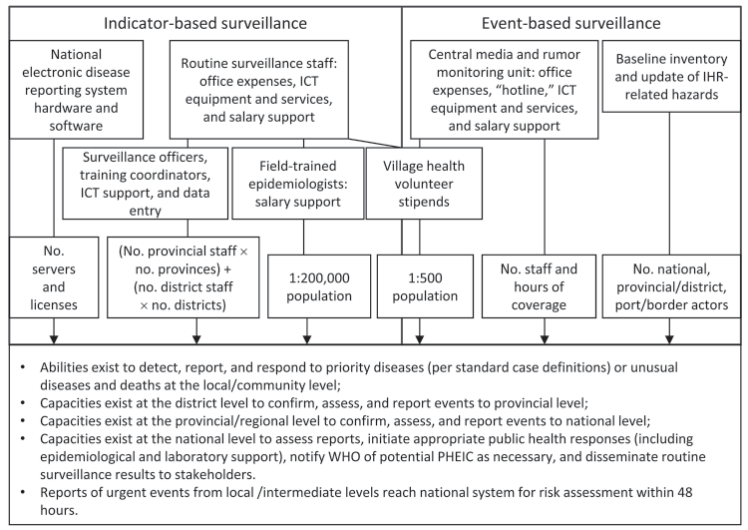
Inputs for Core Capacity 3 (Surveillance). IHR, International Health Regulations; ICT, information and communications technologies; WHO, World Health Organization; PoE, points of entry; PHEIC, public health emergency of international concern.

The IHR Monitoring Framework specifies that this core capacity encompasses indicator-based surveillance (the routine reporting of diseases or syndromes that meet specific case definitions) and event-based surveillance (the rapid detection and reporting of unusual or unexpected disease patterns, deaths, and exposure risks) (Figure 2; Technical Appendix Table 2). All case-study countries conduct national indicator-based surveillance for priority diseases and have developed strategies for combining routine surveillance data with reports from other sources to provide early warning of emerging public health events. The resources for detecting, reporting, and managing cases of priority diseases and unusual events overlap substantially in the Country X template, particularly at the community level.

For Country X, we developed a template for surveillance staffing structure based on a combination of health systems structure and population. We did not attempt to prorate the share of office space, utilities, transportation, etc., dedicated to surveillance for epidemic-prone or emerging infections versus other goals (such as tracking nonommunicable conditions or high-risk behavior).

#### Indicator-based Surveillance

The template for surveillance core capacities in Country X includes activities to support indicator-based surveillance at the central, intermediate, and peripheral levels of the national health system and includes additional capabilities for collecting and analyzing urgent reports for event-based surveillance. Figure 2 provides an overview of Country X inputs; Technical Appendix Table 2 provides a more detailed examination of the infrastructure, human capabilities, and tools and resources for supporting these inputs, including estimates intended to illustrate the approximate costs of implementing IHR core capacities according to this template.

The template uses the proposed minimum standard endorsed by the US Centers for Disease Control and Prevention: 1 field-trained epidemiologist per 200,000 population (*11*). To identify the full operating costs, the template assumes that Country X has achieved this population-based target (300 epidemiologists).

For Country X, numerous provinces with populations of ≈1 million persons serve as the hub for surveillance activities at the intermediate level, and districts (with catchment populations of ≈100,000) serve as the central hubs for surveillance activities at the peripheral level. The template for Core Capacity 3 includes dedicated personnel at the intermediate and peripheral levels to compile and report data on priority diseases and unusual events, to use and disseminate data and guidance issued from the national level, and to train local stakeholders. The template assumes that these functions are housed within existing health offices and health care facilities at the provincial and district levels. We developed the staffing model at each level on the basis of case-study country practices considered by interviewed experts to adequately serve case load.

According to experiences in the case-study countries, some mechanism is needed to extend government disease prevention and control programs to the community level. The inputs for the surveillance template include on-site training of community health center staff and for information and communications technologies equipment and services to facilitate the exchange of information between district health offices, basic health facilities, and communities. The template also includes monthly allowances for training, travel, and communications for 1 village or community health worker per 500 population (the approximate median among the case-study countries with established community health worker networks) to extend disease surveillance and prevention efforts to the household level.

#### Event-based Surveillance

We assume that the infrastructure, capabilities, systems, and processes developed for routine or indicator-based surveillance will also serve as the backbone for event-based surveillance, particularly at the community level where community health volunteers are likely to play a role in collecting reports of unusual disease clusters. The Country X template includes inputs for a center open 24 hours per day, 7 days per week, 365 days per year that would monitor and respond to urgent inquiries from health workers and the public and would collect and analyze structured and unstructured reports. To maximize the use of limited resources, the Country X event monitoring center shares physical infrastructure, information and communications technology resources, and other utilities with the Command and Control Center described in Core Capacity 4.

#### Hazards Mapping

The Country X template includes inputs to develop a baseline inventory of community and national health risks. It applies across all sectors through consultative workshops and field assessments.

### Core Capacity 4: Response

Country X template inputs for response include a functional, dedicated command and control center with room and information and communications technology equipment and services to accommodate up to 40 personnel during an event (Technical Appendix Table 3). Other inputs include materials, supplementary compensation, training, and travel allowances for 2 trained, 5-member multidisciplinary rapid response teams per province, plus 2 central rapid response teams, to investigate and respond to at least 1 public health event per year, with logistical and risk communications support from the provincial level. Inputs also include development and dissemination of guidance for infection control and case management, related training, and systems for isolating and transporting potentially infectious patients.

### Core Capacity 5: Preparedness

The inputs for the Country X preparedness template encompass development, planning, and testing of a national public health emergency response plan (Technical Appendix Table 4). This template is based on a comprehensive national risk assessment and on establishment of a national stockpile of materials to respond to priority events.

### Core Capacity 6: Risk Communications

Inputs for risk communication include the development, printing, and dissemination of a national risk communications plan (Technical Appendix Table 5). These activities are supported by annual training workshops and purchase of broadcast and print media at the peripheral, intermediate, and central levels.

### Core Capacity 7: Human Resources

The inputs for human resources in Country X include resources for developing a coordinated national strategy for public health workforce development (Technical Appendix Table 6). The inputs also include resources for fully supporting field epidemiology training (including travel support for field investigations) for as many as 16 trainees per year.

### Core Capacity 8: Laboratories

For diagnostic testing capabilities, we identified the testing platforms, materials and reagents, and specimen collection and referral systems necessary to support detection and confirmatory testing of the Country X priority diseases as appropriate at each level of a tiered, integrated health system. Technical Appendix Table 7 represents costs only of infrastructure and human capabilities associated with laboratory capacity.

### Points of Entry

The inputs for the Country X template include a health office at each designated point of entry (Technical Appendix Table 8). Each office is staffed by 4-person multidisciplinary public health response teams trained and equipped to respond to medical emergencies.

## Discussion

This study was designed to help decision makers understand the demands of implementing IHR (2005) and to build the business case for strengthening global capacities to detect, assess, report, and respond to public health emergencies. The lack of standards in many areas of public health capacity-building and the many options at almost every step of investment allow for dozens of variations in each category of core capacity, with concomitant variations in costs. However, the framework described in this article illustrates the scope of IHR implementation demands and is intended to help national and international decision makers understand the inputs and associated costs of implementing the IHR (2005).

The framework includes all inputs and associated costs of building capacity for IHR-relevant public health surveillance and response rather than the marginal costs of adding new features to existing surveillance capabilities. Many countries will build the necessary capacities incrementally, and most already have capacities in place. This framework presents a way to estimate one-time capital costs, plus recurrent costs calculated on an annual basis, assuming that the total costs of national implementation depend on variables such as population, existing infrastructure, and health status. For most countries, the first step in developing a national IHR action plan is assessing the gap between current status and their ultimate strategies for implementing IHR core capacities fully.

We believe that this framework serves as a first step in helping national health authorities define to their own governments the actions and investments required to meet their IHR obligations, to protect their populations during public health emergencies, and to build a business case for potential donors. Articulating the elements of IHR core capacity-building can also help the global health community better comprehend a complex obligation that, if implemented fully, will strengthen the public health diagnostic, analytical, and information-sharing capacities that underpin effective decision making across health systems.

## Supplementary Material

Technical Appendix Summaries of inputs and costs for core public health capacities for a model Southeast Asian country with a population of 60 million.
