# Supplementary material for: Costing Framework for International Health Regulations (2005)
Source: Emerg Infect Dis. 2012 Jul;18(7):1121–7. doi: 10.3201/eid1807.120191 (PMC3376815; doi:10.3201/eid1807.120191)
Supplement: Technical Appendix — Summaries of inputs and costs for core public health capacities for a model Southeast Asian country with a population of 60 million. [file 12-0191-Techapp_6p.pdf]

Article DOI: http://dx.doi.org/10.3201/eid1807.120191

Costing Framework for International Health Regulations (2005)

Technical Appendix

Technical Appendix Table 1. Coordination and NFP Communications – Summary of Inputs

Functional capabilities	Inputs	Variables
Protocols and bodies to coordinate stakeholders across sectors are established and tested	Printing and disseminating national IHR action plan and protocols;<br>Convening annual multi-sectoral workshop on IHR implementation	Number of stakeholders;<br>costs of printing, venues, and travel allowances
The designated IHR NFP communicates with WHO and national stakeholders	Office expenses, ICT equipment and access, transportation, and salary support for NFP staff	Number of senior and support staff assigned to NFP functions; salary and communications costs

Technical Appendix Table 2. Summary of Inputs and Costs for Core Capacity 3, Surveillance in Model Southeast Asian Country

IHR Component	Level	Inputs	Description of functions and capacities	Costs in $US 2005
Indicator based, or routine, surveillance	Physical Infrastructure			
	Peripheral: District (600)	Start-up: ICT and office equipment	Per District Health Office plus District Medical Center:<br>- office equipment<br>- communications (fax/phones)<br>- 2 computer stations with UPS	$6414/district<br>$3,848,430 total
		Operating (annual): ICT services, office supplies	Telephone and internet services, mobile phone handsets and usage,	$8357/district<br>$5,014,200 total
	Intermediate: Provincial (64)	Start-up: ICT and office equipment	Per Provincial Health Office plus Provincial Medical Center:<br>- office equipment<br>- communications (fax/phones)<br>- 7 computer stations with UPS	$19,337/province<br>$1,237,579 total
		Operating (annual): Office supplies, ICT, and travel (4 oversight trips to each district)	Telephone and internet services, mobile phone handsets and usage, vehicle fuel/maintenance	$9315/province<br>$596,129 total
	National	Start-up: Hardware for national electronic disease reporting system	Data server, controller and operating system, storage, UPS	$13,005
		Operating (annual): Software licenses/upgrades	Analytical and visualization software	$9,688
	Human Capabilities			
	Peripheral: Sub-district	Operating (annual): Community mobilization and community-based surveillance	Monthly allowance for travel, communications, training for 1 village or community health volunteer per 500 population	$2,880,000–$28,800,000[1]
	Peripheral: District (600)	Operating (annual): Salary support for surveillance staff at district health office and district hospital	Per district:<br>1 surveillance officer (public health assistant equivalent)<br>2 data entry clerks	$10,943,400–$21,777,600 [2]
	Intermediate: Provincial (64)	Operating (annual): Salary support for surveillance staff at provincial health office and provincial hospital	Per province:<br>3 surveillance officers (public health assistant equivalent)<br>1 nurse/trainer<br>1 IT manager<br>2 data entry clerks	$3,024,064–$6,017,536[2]
	National	Operating (annual): Salary support for 300 field-trained epidemiologists assigned to posts nationwide	1 field-trained epidemiologist (public health specialist equivalent) per 200,000 population	$3,522,900–$7,010,400[2]

IHR Component	Level	Inputs	Description of functions and capacities	Costs in $US 2005
	Tools and Processes			
	National	Start-up: Adapt and install national electronic disease reporting system over 12 months	1 full time IT consultant to install and troubleshoot software, integrate maps, standardize databases, train staff	$139,500
		Operating (annual): Update and maintain national electronic disease reporting system	Routine upgrading and maintenance of system hardware and software by IT professional	$18,838
	National	Operating (annual): Travel allowances for one training/oversight visit at each level (national to provincial, provincial to district, district to average of 10 community health centers)	Annual "train-the-trainer" events to review notifiable diseases, case definitions, reporting protocols, strategies	$90,136
Event-based surveillance	Human Capabilities			
	National	Operating (annual): Salary support for 4-person teams to monitor and respond to urgent reports (national hotlines and other sources) on a 24/7/365 basis	Per team (x 4):<br>1 public health specialist<br>3 public health assistants	$134,008–$266,656[2]
Surveillance overview of information on IHR related hazards (situation awareness)	Human Capabilities			
	National	Start-up: Assessment of public health risks across all sectors nationwide	One short-term consultant to coordinate data collection and develop database	$23,250
	Tools and Processes			
	National	Operating (annual): Review and update data on all health hazards with public and private sector stakeholders	Annual consultative workshop on IHR-relevant risks	$4,920

[1] Village/community health worker stipends vary from $20/year to $20/month among case study countries, reflected here as a range of national-level costs.
[2] Range reflects variation in average salaries for equivalent positions between WPRO B and SEARO B sub-regions in WHO CHOICE dataset.

Technical Appendix Table 3. Summary of Inputs and Costs for Core Capacity 4, Response in Model Southeast Asian Country

IHR Component	Level	Inputs	Description of functions and capacities	Costs in $US 2005
Rapid response capacity	Physical Infrastructure			
	Intermediate: Provincial (64)	Start-up: Dedicated transport for Rapid Response Teams	One vehicle for each Rapid Response Team (RRT) (2 per province plus 2 central)	$2,591,550
		Operating (annual): Consumables for community-level outbreak investigation	2 outbreak investigation kits (specimen collection, packaging, record-keeping and PPE), per WHO checklist, plus vehicle fuel and maintenance	$1,253,492
	National	Start-up: Dedicated Command and Control Center – facility plus ICT equipment and services for up to 40 personnel during a crisis	Construction (400 m^2) plus ICT equipment (including satellite phones, radios, videoconferencing) and standby generator	$967,782
		Operating (annual): ICT services	Routine use by full-time (call and event monitoring staff) plus surge capacity	$23,820
	Human Capabilities			
	Intermediate: Provincial (64)	Operating (annual): Supplementary compensation for RRT members and full-time salary support for financial, logistical, risk communications, and training staff	Per RRT -- bonuses (equivalent to 1 week's annual salary) for:<br>1 Epidemiologist<br>1 Laboratory Specialist<br>1 Medical Officer<br>1 Public Health Specialist<br>1 Animal and/or Environmental Health Specialist; and<br>Full salary support for one finance officer, one administration officer, and one risk communications officer per province	$1,247,013–$2,481,576[2]

IHR Component	Level	Inputs	Description of functions and capacities	Costs in $US 2005
	Tools and Processes			
	National	Operating (annual): Travel allowances for all RRTs plus for field investigations	Annual national training conference for all Rapid Response Team members and travel allowances for outbreak investigations (1 week per team member per year)	$443,230
Case management	Physical infrastructure			
	Intermediate: Provincial (64)	Start-up: Patient transport system and equipment	Ambulances for patient transport (average of 2.5 per province nationwide)	$8,000,000
		Operating (annual): Annual vehicle maintenance costs	Maintenance for 2.5 ambulances per province	$775,000
Infection prevention and control	Physical Infrastructure			
	Intermediate: Provincial (64) and District (600)	Start-up: 2 airborne infection isolation (negative pressure) rooms per province and 1 respiratory isolation unit (multi-bed) per district	Construction/renovation costs for 2 isolation rooms per province and conversion costs for one room per district	$8,921,000
		Operating (annual): Consumables for isolation units	Annual HEPA filter changers per isolation room	$130,405
	Human Capabilities			
	National	Operating (annual): National Infection Prevention and Control program and training coordinator	Salary support and travel allowances for one program coordinator and one trainer	$28,398
	Tools and Processes			
	National	Operating (annual): Training and oversight for Infection Prevention and Control at facility levels	Printing and dissemination of protocols and annual oversight visits at the provincial level	$41,936

[1] Village/community health worker stipends vary from $20/year to $20/month among case study countries, reflected here as a range of national-level costs.
[2] Range reflects variation in average salaries for equivalent positions between WPRO B and SEARO B sub-regions in WHO CHOICE dataset.

Technical Appendix Table 4. Summary of inputs and costs for Core Capacity 5 (Preparedness) in Model Southeast Asian Country

IHR Component	Level	Inputs	Description of functions and capacities	Costs in $US 2005
Risk and resource management	Physical Infrastructure			
	National	Start-up: Climate controlled stockpile storage facility and management	1000 m2 warehouse (new construction) with air conditioning and generator; 2 delivery vehicles; inventory management software and computer	$2,814,166
		Operating (annual): Medical equipment, personal protective equipment, and consumable supplies	Scenario-based estimates for surge capacity to accommodate 100 patients of severe/moderate acuity for 14 days plus equipment and supplies for 25 patients with novel influenza per province	$1.6 M/province<br>$103.5M total
	Human Capabilities			
	National	Operating (annual): Salary support for 3-person stockpile management staff plus 24/7/365 security coverage	Manager<br>Finance officer<br>Transport driver/porter<br>Security guard (4 shifts)	$33,811–$67,729
Public health emergency preparedness and response plan	Human Capabilities			
	National	Operating (annual): Salary support for national emergency exercise staff	1 program manager<br>2 nurse/trainers	$26,249–$52,232
	Tools and Processes			
	National	Operating (annual): Planning, implementation, and oversight for 1-day annual table-top exercise at the national, provincial, and district levels:	Supervisory visits to support planning, execution, and reporting of exercises<br>- National to provincial (2 trainers x 3 days)<br>- Provincial to district (1 trainer x 2 days)<br>Printing and dissemination of exercise materials and after action reports for participants<br>Travel and meeting expenses (100 participants each at the national and provincial levels and 20 participants at each district level)	$95,948

Technical Appendix Table 5. Summary of inputs and costs for Core Capacity 6, Risk Communication in Model Southeast Asian Country

IHR Component	Level	Inputs	Description of functions and capacities	Cost in $US 2005
Policy and procedures for risk communications	Human Capabilities			
	National	Start-up: Salary support a public health specialist to oversee development of national risk communication plan	Full-time salary equivalent for one public health specialist for 3 months	$4,389
	Tools and Processes			
	National	Operating (annual):Consultative annual meeting for stakeholders to develop community-based national risk communication plan.	Per diem allowance for 50 stakeholders, and printing and dissemination of plan	$4,367
	National	Operating (annual): Annual national workshop for risk communication stakeholders	Per diem and travel allowances for stakeholders at provincial and district level, and printing of training materials	$87,258
	National	Operating (annual): Broadcast and print media budget	Television, radio, and newspaper announcements for contingency messages, and posters/flyers for routine messages.	$1,777,244–2,050,314

Technical Appendix Table 6. Summary of inputs and costs for Core Capacity 7 (Human Resources) in Model Southeast Asian Country

IHR Component	Level	Inputs	Description of functions and capacities	Costs in $US 2005
Policy and training for workforce development	Human Capabilities			
	National	Start-up: Salary support for staff to oversee development of public health workforce plan (part-time)	1 public health specialist (equivalent to 3 months' effort)	$2,936-$5,842
	Tools and Processes			
	National	Operating (annual): Review, revise, and disseminate public health workforce plan as needed	Two 1-day consultative meetings of 25 national stakeholders to review workforce development plan; printing and dissemination of plan	$8,389
		Operating (annual): Field Epidemiology Training Program (FETP) administrative costs plus stipends and travel allowance for 16 fellows total each year	Salary support for<br>FETP program manager<br>Administrative/logistical staff<br>Training stipends for 8 fellows (2 cohorts per year at the salary equivalent of public health specialist (total of 16 fellow in two-year program)<br>Per-fellow allowance for travel, supplies, and equipment, assuming at least 1 field investigation/year	$612,260–$644,620

Technical Appendix Table 7. Summary of Inputs and Costs for Core Capacity 8, Laboratory in Model Southeast Asian Country

IHR Component	Level	Inputs	Description of functions and capacities	Cost in $US 2005
Laboratory diagnostic testing	Physical Infrastructure			
	National	Start-up: Construction of laboratories plus data management office	7 work spaces: specimen processing, clean prep, BSL2 and BSL3 virology, BSL2 and BSL3 microbiology, electrophoresis and visualization	$865,961
		Operating (annual): Utilities for national laboratories	Costs of water and energy services; laboratory equipment inventory and condition	$57,288
	Provincial	Start-up: Laboratory construction	64 provincial labs at an average of $615,545 per site. No BSL3 work spaces; specimen processing, clean prep, BSL2 virology, BSL2 microbiology, electrophoresis and visualization	$39,394,880
	National, provincial, and district	Start-up: Voltage stabilizers and air conditioners plus standby generators	Equipment for labs at all , except generators which will be required only at national and provincial levels. National labs, 64 provincial labs, and 600 district labs.	$892,472

IHR Component	Level	Inputs	Description of functions and capacities	Cost in $US 2005
	Provincial and district	Operating (annual): Utilities for provincial and district laboratories	Costs of water and energy services; equipment inventory and condition; # of labs	$5,000,000
	Human Capabilities			
	National	Operating (annual): Salary support for laboratory staff in national laboratories	Each virology and microbiology laboratory will include 1 senior program manager, 2 laboratory specialists, 2 laboratory technologists, and 2 laboratory assistants each, plus administrative and maintenance support	$169,374–$337,038
		Operating (annual): Salary support for national quality resource center staff	Annual salaries for national quality resource center director and trainers and procurement director	$37,992–$75,600
	Provincial	Operating (annual): Salaries for virology and microbiology laboratory staffs in 64 provincial laboratories	Virology and microbiology laboratory team will contain 1 manager, 1 specialist, 2 assistants each, plus administrative and maintenance support	$5,579,072–$11,101,568
	District	Operating (annual): Salary support for laboratory staff in 600 district labs	Each district lab will have 1 laboratory technologist and 1 assistant per lab	$18,996–$37,800
	Tools and Processes			
	National	Operating (annual): Guidance and training on national surveillance, laboratory, and specimen referral strategies	Travel allowance for staff at provincial and district level and costs of printing materials and printing costs; # of staff at sub-national level	$41,874
		Operating (annual): National EQAS documentation, guidance, and oversight visits	Visits to sub-national labs subject to oversight	$16,054
Specimen collection and transport	Physical Infrastructure			
	Provincial	Operating (annual): Specimen transport	2 dedicated vehicles per province for 64 provinces with cold boxes	$2,686,976
	Human Capabilities			
	Provincial	Operating (annual): Salary support for administrative staff	2 transport drivers per province for 64 provinces	$539,264–$1,073,024
	Tools and Processes			
	National	Operating (annual): shipment of infectious agents	10 expedited international shipments at the national level and 10 expedited domestic shipments at the provincial level (10 per province) with complete packaging	$125,345
	National and provincial	Operating (annual): Training and documentation in shipment of infectious agents for laboratory staff	Strengthening and maintaining domestic diagnostic confirmation capabilities, accessibility of courier services, and specimen referral protocols	$35,770
Laboratory biosafety	Physical Infrastructure			
	National, provincial, and district	Start-up: Laboratory equipment	Biosafety cabinets (class II) and large autoclaves at national and provincial levels, and mid-sized autoclaves at each district site	$4,897,561
		Operating (annual): Maintenance and certification of laboratory equipment	Annual maintenance and certification of biosafety cabinets at national and provincial levels	$35,770
	National	Start-up: National biosafety training facility	Central training facility	$139,682
	Human Capabilities			
	National	Operating (annual): Salary support for staff	Salaries for biosafety officers and trainers	$35,229–$70,104
	Tools and Processes			
	National	Operating (annual): Annual training of stakeholders	Biosafety training, guidance and documentation	$60,780
	National, provincial, and district	Operating (annual): Supplies and equipment for biosafety laboratories	Sharps containers, biohazard bags, and personal protective equipment for laboratories at all levels	$1,537,911
Laboratory-based surveillance	Physical Infrastructure			
	National, provincial and district	Start-up: ICT equipment for management and administration of daily activities	ICT: phone, fax, computers, printers, GPS	$602,228

IHR Component	Level	Inputs	Description of functions and capacities	Cost in $US 2005
	National, provincial and district	Operating (annual): ICT access for surveillance and data management	Phone, mobile, and internet access and management	$441,721
	Human Capabilities			
	National	Operating (annual): Salary support for administrative staff	Salary for one data entry clerk	$8213–$9572
	Tools and Processes			
	District	Operating (annual): Reporting system	Paper-based weekly reporting forms for district labs	$2,040

Technical Appendix Table 8. Summary of inputs and costs for Points of Entry in Model Southeast Asian Country

IHR Component	Level	Inputs	Description of functions and capacities	Costs in $US 2005
Emergency response units at Points of Entry	Physical Infrastructure			
	National	Start-up: Transportation for ill passengers from plane or health unit to nearest appropriate healthcare facility (plus annual maintenance) for six points of entry sites	One vehicle per Point of Entry site to supplement existing ambulance capacity	$148,673
		Operating (annual): Health units at Points of Entry for six points of entry sites	Real property rental, information and communications technology, office supplies, office equipment and supplies	$115,200
	Human Capabilities			
	National	Start -up: Salary support for staff to develop national contingency plan	1 public health consultant for 3 months	$4,389
		Operating (annual): Salary support for Points of Entry Response Teams with 24/7/365 coverage at six sites and two annual oversight visits per site	1 medical officer<br>1 nurse<br>1 public health assistant<br>1 administrative assistant/IT officer<br>Exenses for 12 oversight visits annually	$604,015–$1,200,931
	Tools and Processes			
	National	Operating (annual): One-day workshop of stakeholders from relevant ministries and provincial/regional health facilitates to review the national public health emergency response plan , and Outbreak investigation/response kids for six sites	Printing and dissemination of plan<br>Per diem for stakeholders<br>2 response kits per site (total of 12)<br>PPE<br>Decontamination material<br>Diagnostic/treatment supplies	$119,676
